# Asymmetry analysis of optical coherence tomography angiography macular perfusion density measurements in preperimetric and perimetric glaucoma

**DOI:** 10.1038/s41598-020-71757-6

**Published:** 2020-09-08

**Authors:** Pei-Yao Chang, Jiun-Yi Wang, Jia-Kang Wang, Shih-Cheng Yeh, Shu-Wen Chang

**Affiliations:** 1 Department of Ophthalmology, Far Eastern Memorial Hospital, Ban-Chiao, New Taipei City, Taiwan, ROC; 2 Department of Medicine, National Taiwan University Hospital, Taipei, Taiwan, ROC; 3 Department of Healthcare Administration and Department of Nursing, Oriental Institute of Technology, New Taipei City, Taiwan, ROC; 4 Department of Electrical Engineering, Yuan Ze University, Taoyuan City, Taiwan, ROC; 5 Department of Healthcare Administration, Asia University, Taichung, Taiwan, ROC; 6 Department of Medical Research, China Medical University Hospital, China Medical University, Taichung, Taiwan, ROC; 7 Department of Statistics and Information Science, Fu Jen Catholic University, New Taipei City, Taiwan, ROC

**Keywords:** Biomarkers, Diseases

## Abstract

Macular retinal layer thickness asymmetry indices, particularly for the ganglion cell layer, are promising early indicators of glaucomatous damage. We evaluated macular perfusion density asymmetry (MPDA) among normal, preperimetric glaucoma (PPG), and perimetric glaucoma (PG) eyes, and we tested the performance of MPDA in differentiating between control and glaucoma eyes with or without visual field (VF) defects. In this study, 116 eyes (39 normal, 27 PPG, and 50 PG eyes) with optical coherence tomography angiography images of the macula were analysed. No significant difference was found in outer and inner MPDA between the control and PPG groups. However, outer MPDA was significantly higher in the PG group than in the PPG group (p = 0.009). Asymmetry of perfusion density and structural parameters was compared; no significant difference was found between controls and glaucoma patients. Outer MPDA had significantly higher discrimination ability between PPG and PG than did macular ganglion cell layer–inner plexiform layer thickness asymmetry (p = 0.039). In conclusion, the discriminant capability of MPDA for discriminating between glaucoma patients with and without VF defects is significantly higher than that of structural asymmetry. MPDA may be helpful in monitoring glaucoma progression in clinical practice.

## Introduction

Evaluation of the circumpapillary retinal nerve fibre layer (cpRNFL) is useful for glaucoma diagnosis; however, more than 50% of retinal ganglion cell somas are located within the macula. Increasing evidence implicates early macular involvement in glaucomatous damage^1–3^, and it has been highlighted that cpRNFL analysis alone is insufficient for detecting macular damage^4^. Kim et al.^2^ reported that macular ganglion cell layer–inner plexiform layer (mGCIPL) changes are detected even before corresponding cpRNFL changes in the optical coherence tomography (OCT) deviation map.

Optical coherence tomography angiography (OCTA) is a newly developed noninvasive imaging modality that provides for qualitative and quantitative evaluation of the microvasculature of different retinal layers. Researchers have used OCTA to quantify the density of perfused vessels in peripapillary and macular regions in glaucoma patients, and increasing evidence has shown strong correlations between vessel density measurements and cpRNFL defects, visual field (VF) deficits, and glaucoma severity^5–8^. Studies have even revealed that OCTA vascular density parameters have stronger correlations with VF defects than do OCT structural parameters^7,8^.

The thicknesses of the cpRNFL, mGCIPL, and ganglion cell complex (GCC) in normal eyes are highly symmetrical in the upper and lower retinal hemispheres^9,10^. The glaucoma hemifield test (GHT) of the VF, which measures disparity in retinal sensitivity between the superior and inferior hemifields, is regarded as a useful tool in glaucoma assessment^11,12^. Macular retinal layer thickness asymmetry indices, particularly for the ganglion cell layer, are promising early indicators of glaucomatous retinal damage^10,13^. However, limited information is available on differences in superficial macula microvasculature damage between superior and inferior hemifields in glaucoma patients. It is desirable to have asymmetry indices in OCTA measurement for the early detection of glaucomatous change. Therefore, this study evaluated differences in hemifield macula vascular density among normal, preperimetric glaucoma (PPG), and perimetric glaucoma (PG) groups and tested its performance in distinguishing between controls and glaucoma patients with or without VF defects.

## Methods

This retrospective cross-sectional study was conducted between January and December 2018. Patients with primary open-angle glaucoma (POAG) and healthy individuals (control group) who visited the glaucoma clinic of Far Eastern Memorial Hospital were recruited. The study protocol adhered to the tenets of the Declaration of Helsinki and was approved by the Institutional Review Board and Ethics Committee of Far Eastern Memorial Hospital, which waived the need for informed consent, as this study was based on retrospective data.

The inclusion criteria for all groups were as follows: age more than 20 years, best-corrected visual acuity ≥ 20/30, and refractive errors >  − 6 diopters. Patients with systemic hypertension and diabetes mellitus were included unless they had hypertensive or diabetic retinopathy. The exclusion criteria for all groups were as follows: presence of any other retinal or optic nerve disease, previous ocular surgery, or presence of any media opacities that prevented high-quality OCT scans. Diagnostic criteria for glaucoma were a normal anterior segment in slit-lamp examination, an open angle in gonioscopy, presence of glaucomatous optic nerve head changes (i.e., neuroretinal rim thinning, notching, cupping, and optic disc haemorrhage), or an RNFL defect in red-free fundus photography^6^. Eyes with glaucomatous VF defects were defined as those with a GHT result outside normal limits or a pattern standard deviation outside 95% of normal limits. Additionally, a cluster of three points with probabilities of 5% on the pattern deviation map in at least one hemifield, including at least one point with a probability of 1%, or a cluster of two points with a probability of 1% was needed^10^. To include preperimetric glaucoma eyes, VF defect was not considered as a diagnostic criterion in the present study.

Each subject underwent complete ophthalmic examination that included the following assessments: slit lamp biomicroscopy, intraocular pressure (IOP) measurement through noncontact tonometry, refractive error measurement through autorefraction (Auto Refractometer AR-610; Nidek Co, Ltd., Tokyo, Japan), gonioscopy, and dilated fundus examination with simultaneous stereophotography of the optic disc and red-free RNFL photography. VF testing was performed using a Humphrey Field Analyzer (SITA full threshold programs 30-2; Carl Zeiss Meditec, Inc., Dublin, CA, USA).

**Figure 1:**
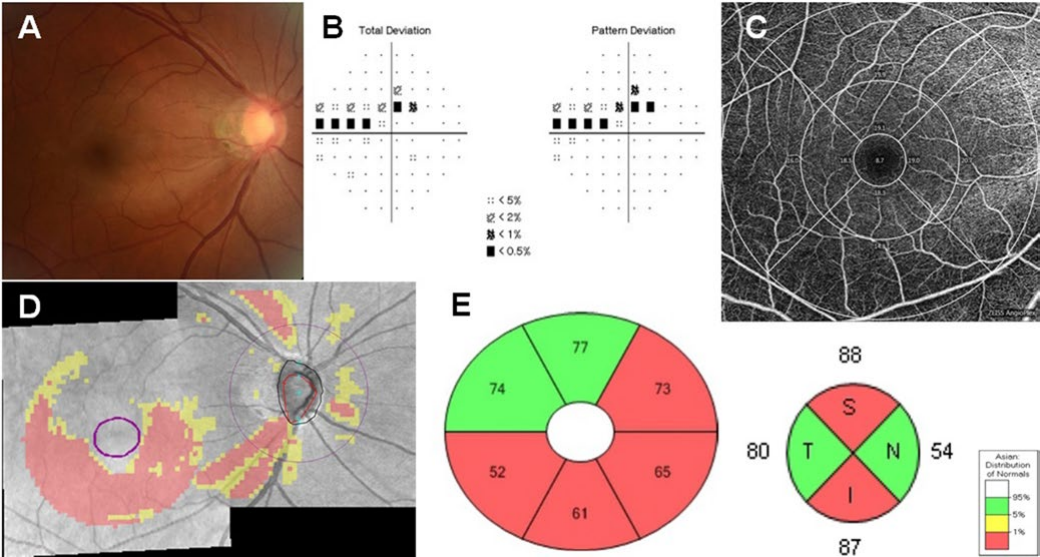
Inferior disc excavation with inferior nerve fibre layer wedge defect of right eye of a 52-year-old woman with POAG (**a**). Visual field results revealed defects at the corresponding superonasal site (**b**). OCT angiography image (Cirrus HD-OCT , version 10.0.0.14618) of a 6 × 6 mm scan exhibited the superficial vessels at the macula. The grids represented the sectors across which the vessel length densities (/mm) (**c**) were calculated. The area between the inner two circles in the 6 × 6 mm scan represented the inner sectors, and the area between the outer two circles represented the outer sectors. Combined RNFL and GCIPL deviation maps indicated structural glaucomatous damage (**d**). OCT scan showed the GCIPL thickness (μm) provided by the ganglion cell analysis report (**e**, left) and RNFL thickness (μm) (**e**, right) in each sector. *GCIPL* ganglion cell-inner plexiform layer; *OCT* optical coherence tomography. *I* inferior; *IN* inferonasal; *IT* inferotemporal; *N* nasal; *S* superior; *SN* superonasal; *ST* superotemporal; *T* temporal.

Peripapillary (Optic Disc Cube 200 × 200 protocol) and macular (Macular Cube 512 × 128 protocol) scans (collectively referred to as ganglion cell analysis) were acquired using the Cirrus 5000 HD-OCT (Carl Zeiss Meditec, Inc.). Software released by the manufacturer was used to calculate RNFL and GCIPL thicknesses, as previously described^14^. OCTA imaging of the macula was performed using the Cirrus HD-OCT (version 10.0.0.14618). The procedure for OCTA imaging using the Cirrus HD-OCT has been detailed previously^15^. Angiographic images were generated through OCT-based microangiography (OMAG), and the macula was imaged using a 6 × 6 mm^2^ scan pattern. Angiometric software of the Cirrus HD-OCT automatically calculates two parameters from the superficial retinal layer slab: vessel length density (defined as the total length of perfused vasculature per unit area in the region of measurement) and perfusion density (defined as the total area of perfused vasculature per unit area in the region of measurement). This software calculates the vessel length and perfusion density parameters across four inner and four outer sectors of the Early Treatment Diabetic Retinopathy Study (ETDRS) grid over the macula (Fig. 1). Image quality was assessed for all OCTA and OCT scans. Poor quality images, which were defined as those with a signal strength of < 7, poor centration, or motion artefacts and segmentation errors, were excluded from analysis. Outer macular perfusion density asymmetry (MPDA) was defined as the absolute value of the difference between outer superior and outer inferior parafoveal perfusion density, and the same definition was applied to inner MPDA. Outer macular vessel length density asymmetry (MVDA) was defined as the absolute value of the difference between outer superior and outer inferior parafoveal vessel length density, and the same definition was applied to inner MPDA. For mGCIPL asymmetry, it was defined as the absolute value of the difference between superior and inferior mGCIPL thicknesses; average mGCIPL asymmetry was defined as the absolute value of the difference between the average superior (i.e., superior + superotemporal + superonasal mGCIPL thicknesses/3) and average inferior (i.e., inferior + inferotemporal + inferornasal mGCIPL thicknesses/3) mGCIPL thicknesses. For cpRNFL asymmetry, it was defined as the absolute value of the difference between superior and inferior cpRNFL thicknesses.

### Statistical analysis

Descriptive statistics included mean and standard deviation for normally distributed variables and median and interquartile range for nonnormally distributed variables. The Shapiro–Wilk test was used to examine the normality distribution of continuous variables. If both eyes of a patient met the inclusion criteria, one eye was randomly selected. Depending on data distribution, ANOVA or the Kruskal–Wallis test with a post hoc test was conducted to compare baseline characteristics. The area under the receiver operating characteristic curve (AUC) was used to evaluate the diagnostic ability of asymmetry of OMAG and structural thickness parameters between the groups. A comparison of two AUCs was conducted in the package of pROC in R-language with the bootstrap method^16^. For each parameter of the direct measurement and asymmetry of OMAG and structural thickness, three post hoc tests or pairwise comparisons were conducted, and the significance level was set at 0.017 according to Bonferroni correction^17^. For other tests, a p value of < 0.05 was considered statistically significant. Due to the limited sample size available in the hospital, G-Power 3.1 was used to estimate the power for critical parameters given the observed sample size. Powers of 99.7%, 99.5%, and 61.4% were estimated for outer MVDA, outer MPDA, and average mGCLPL thickness asymmetry, given the sample sizes were 27 and 50 for the PPG and PG groups, respectively, at the significance level of 0.017, and the estimated effect sizes based on the observed data.

## Results

In total, 151 individuals (50 normal and 101 POAG patients) underwent OCTA measurements of the macula. Among them, 30 eyes (19.9%) with motion artefacts in OMAG scans and 4 eyes (3.31%) with media opacity such as vitreous floaters were excluded. Finally, 116 individuals (116 eyes), comprising 39 healthy subjects, 27 PPG patients, and 50 PG patients, were included in the analysis. The demographic and ophthalmic characteristics of the three groups are summarised in Table 1. All participants in the glaucoma group were treated with at least one type of ocular antihypertensive medication at the time of OCTA imaging.

**Table 1:** Baseline characteristics of control and glaucoma groups.

	Median (25% Quartile,75% Quartile)			*P*	Post hoc Test		
	Control (n = 39)	PPG (n = 27)	PG (n = 50)		Control vs PPG	PPG vs PG	Control vs PG
Age (y)	51.0 ± 10.7	49.1 ± 12.3	56.5 ± 9.4	0.007	0.770	0.016	0.057
Gender (male:female)	21:18	11:16	36:14	0.02	0.30	0.007	0.08
Eye (OD:OS)	20:19	16:11	16:34	0.05			
Diabetes (yes:no)	3:36	3:24	4:46	0.87			
Hypertension (yes:no)	5:34	4:23	6:44	0.94			
Spherical equivalence (D)	− 1.00 (− 2.50 to 0.25)	− 4.00 (− 5.00 to − 1.50)	− 2.25 (− 4.38 to 0.00)	0.008	0.006	0.28	0.25
Visual field index (%)	99.0 (97.8–100.0)	99.0 (98.0–99.0)	87.5 (75.0–94.3)	< 0.001	1.00	< 0.001	< 0.001
Mean deviation (dB)	− 0.1 (− 2.3 to 0.6)	− 0.5 (− 2.2 to 0.0)	− 4.9 (− 8.1 to − 2.6)	< 0.001	1.00	< 0.001	< 0.001
Optic disc area (${\mathrm{mm}}^{2}$)	2.09 (1.81–2.27)	1.84 (1.72–2.15)	1.93 (1.56–2.22)	0.13			
Rim area (mm)	1.17 (1.11–1.31)	0.97 (0.83–1.13)	0.78 (0.67–0.93)	< 0.001	0.003	0.001	< 0.001
Average C/D ratio	0.65 (0.54–0.71)	0.67 (0.58–0.75)	0.76 (0.69–0.82)	< 0.001	0.26	0.002	< 0.001
IOP at the scanning visit (mm Hg)	17.0 (15.7–20.7)	15.0 (13.8–18.3)	13.7 (12.4–15.6)	< 0.001	0.31	0.03	< 0.001

*PPG* preperimetric glaucoma; *PG* perimetric glaucoma; *IOP* intraocular pressure.

No significant differences were observed in the vessel length or perfusion density in the macular area between the control and PPG groups, regardless of the sector. However, mGCIPL thickness in every sector was significantly lower in the PPG group than in the control group (all p < 0.017). In addition, cpRNFL in the superior and inferior sectors was significantly lower in the PPG group than in the control group (both p < 0.001; Table 2).

**Table 2:** Optical coherence tomography angiography vessel density parameters and structural measurements of participants.

	Median (25% Quartile, 75% Quartile)			Pairwise comparison		
	Control (n = 39)	PPG (n = 27)	PG (n = 50)	Control vs PPG	PPG vs PG	Control vs PG
**Signal strength of 6 × 6 mm OMAG**						
SS (macular scan)	9 (8–10)	9 (8–10)	8 (7–9)	0.759	0.920	0.176
**Macular vessel length density (/mm)**						
Outer superior	18.2 (16.6–19.1)	17.7 (16.4–18.8)	15.3 (13.5–17.7)	0.897	0.124	0.009
Outer inferior	18.1 (16.0–19.2)	16.8 (15.7–17.9)	12.8 (10.1–16.1)	0.093	0.031	< 0.001
Outer temporal	17.2 (14.4–18.3)	15.9 (12.8–17.8)	13.9 (10.9–16.0)	0.885	0.251	0.004
Outer nasal	19.7 (18.7–20.2)	19.8 (18.9–20.4)	18.6 (16.3–19.8)	0.230	0.888	0.184
Inner superior	18.0 (15.1–19.0)	16.8 (15.1–18.8)	16.6 (12.6–17.8)	0.785	0.870	0.246
Inner inferior	18.1 (15.8–19.2)	17.5 (14.4–19.0)	15.4 (12.1–17.2)	0.369	0.559	0.036
Inner temporal	17.4 (15.6–18.8)	17.5 (14.1–19.1)	15.3 (12.7–17.9)	0.531	0.147	0.013
Inner nasal	18.1 (15.3–19.0)	16.3 (14.9–19.4)	17.2 (14.1–18.8)	0.187	0.235	0.710
Inner mean	18.1 (15.6–19.1)	16.4 (15.3–19.1)	16.2 (13.0–17.4)	0.374	0.627	0.029
Outer mean	18.1 (16.7–19.0)	17.7 (16.0–18.4)	15.6 (12.9–17.1)	0.459	0.304	0.006
Full mean	17.5 (16.3–18.8)	17.2 (15.4–18.5)	15.5 (12.8–16.8)	0.217	0.348	0.081
**Macular perfusion density**						
Outer superior	0.45 (0.41–0.47)	0.44 (0.40–0.46)	0.38 (0.34–0.43)	0.681	0.180	0.005
Outer inferior	0.44 (0.39–0.47)	0.42 (0.38–0.44)	0.32 (0.25–0.41)	0.230	0.119	< 0.001
Outer temporal	0.42 (0.33–0.45)	0.38 (0.30–0.44)	0.34 (0.26–0.39)	0.981	0.211	0.005
Outer nasal	0.48 (0.45–0.49)	0.49 (0.47–0.50)	0.45 (0.39–0.49)	0.229	0.763	0.071
Inner superior	0.42 (0.36–0.46)	0.39 (0.35–0.47)	0.39 (0.29–0.44)	0.657	0.492	0.286
Inner inferior	0.43 (0.37–0.46)	0.40 (0.35–0.46)	0.37 (0.28–0.42)	0.561	0.950	0.111
Inner temporal	0.41 (0.37–0.44)	0.39 (0.32–0.45)	0.36 (0.29–0.43)	0.917	0.165	0.144
Inner nasal	0.43 (0.35–0.44)	0.38 (0.35–0.45)	0.41 (0.32–0.45)	0.077	0.148	0.788
Inner mean	0.42 (0.37–0.45)	0.38 (0.35–0.46)	0.38 (0.31–0.42)	0.327	0.609	0.096
Outer mean	0.44 (0.41–0.47)	0.43 (0.39–0.45)	0.38 (0.30–0.42)	0.620	0.431	0.017
Full mean	0.44 (0.40–0.46)	0.41 (0.37–0.45)	0.38 (0.31–0.41)	0.431	0.417	0.076
mGCIPL (μm)*						
Superior thickness	85.1 ± 6.3	77.6 ± 4.8	70.1 ± 9.9	< 0.001	0.006	< 0.001
Inferior thickness	81.6 ± 4.8	71.7 ± 5.7	64.0 ± 10.3	< 0.001	0.004	< 0.001
Superotemporal thickness	83.4 ± 5.7	74.4 ± 5.2	66.1 ± 12.0	< 0.001	0.008	< 0.001
Inferotemporal thickness	83.5 ± 5.0	73.3 ± 6.1	61.4 ± 12.6	< 0.001	< 0.001	< 0.001
Superonasal thickness	86.1 ± 6.8	79.6 ± 7.0	75.1 ± 10.6	0.001	0.068	< 0.001
Inferonasal thickness	84.3 ± 5.5	77.1 ± 5.3	70.8 ± 10.7	< 0.001	0.012	< 0.001
Average superior thickness	84.9 ± 5.9	77.2 ± 4.8	70.4 ± 9.9	< 0.001	0.008	< 0.001
Average inferior thickness	83.1 ± 4.6	74.0 ± 4.5	65.4 ± 10.5	< 0.001	0.001	< 0.001
Average mGCIPL thickness	84.0 ± 5.2	75.6 ± 3.9	67.9 ± 9.5	< 0.001	0.001	< 0.001
cpRNFL (μm)*						
Superior thickness	122.1 ± 12.3	101.7 ± 16.7	90.9 ± 17.8	< 0.001	0.032	< 0.001
Inferior thickness	126.1 ± 12.1	99.7 ± 18.0	78.4 ± 22.1	< 0.001	0.001	< 0.001
Temporal thickness	72.9 ± 13.9	71.0 ± 10.5	60.8 ± 13.7	0.052	0.064	< 0.001
Nasal thickness	71.4 ± 10.6	68.6 ± 9.2	65.0 ± 8.4	0.640	0.033	0.003
Average cpRNFL thickness	99.0 ± 7.6	85.3 ± 6.4	73.8 ± 10.3	< 0.001	0.000	< 0.001

All values represent median with interquartile range in parentheses unless specified * (mean ± SD).

Age, gender, and spherical equivalence were adjusted in pairwise comparisons.

*PPG* preperimetric glaucoma; *PG* perimetric glaucoma; *mGCIPL* macular ganglion cell-inner plexiform layer; *cpRNFL* circumpapillary retinal nerve fibre layer asymmetry; *OMAG* optical microangiography.

Table 3 summarises the asymmetry of OMAG parameters and structural thickness parameters in glaucoma patients and controls. In structural analysis, cpRNFL thickness asymmetry was significantly higher in the PPG group than in the control group (p = 0.007). However, outer and inner MVDA exhibited no significant difference between the control and PPG groups. However, outer MPDA was significantly higher in the PG group than in the control and PPG groups (both p < 0.01).

**Table 3:** Asymmetry of OMAG parameters and structural thickness parameters in glaucoma and control groups.

	Median (25% quartile, 75% quartile)			Pairwise comparison		
	Control (n = 39)	PPG (n = 27)	PG (n = 50)	Control vs PPG	PPG vs PG	Control vs PG
Macular outer vessel length density asymmetry (/mm)	0.7 (0.2–1.4)	1.0 (0.5–1.7)	2.7 (1.7–4.6)	0.580	0.051	< 0.001
Macular inner vessel length density asymmetry (/mm)	0.7 (0.2–1.5)	0.6 (0.4–2.0)	1.5 (0.5–2.5)	0.743	0.528	0.199
Macular outer perfusion density asymmetry	0.02 (0.01–0.03)	0.02 (0.01–0.04)	0.08 (0.05–0.11)	0.582	0.009	< 0.001
Macular inner perfusion density asymmetry	0.02 (0.01–0.04)	0.02 (0.01–0.06)	0.03 (0.01–0.06)	0.865	0.355	0.235
mGCIPL thickness asymmetry (μm)	5.0 (3.0–6.0)	5.0 (3.0–9.0)	8.5 (2.0–14.0)	0.814	0.054	0.004
Average mGCIPL thickness asymmetry (μm)	2.7 (1.7–3.7)	4.0 (1.0–6.0)	7.5 (2.5–11.0)	0.959	0.018	0.001
cpRNFL thickness asymmetry (μm)	7.0(4.0–11.8)	19.0(7.0–33.0)	20.5(11.0–33.3)	0.007	0.366	0.002

All values represent median with interquartile range in parentheses.

Age, gender, and spherical equivalence were adjusted in pairwise comparisons.

*PPG* preperimetric glaucoma; *PG* perimetric glaucoma; *mGCIPL* macular ganglion cell-inner plexiform layer; *cpRNFL* circumpapillary retinal nerve fibre layer asymmetry.

**Figure 2:**
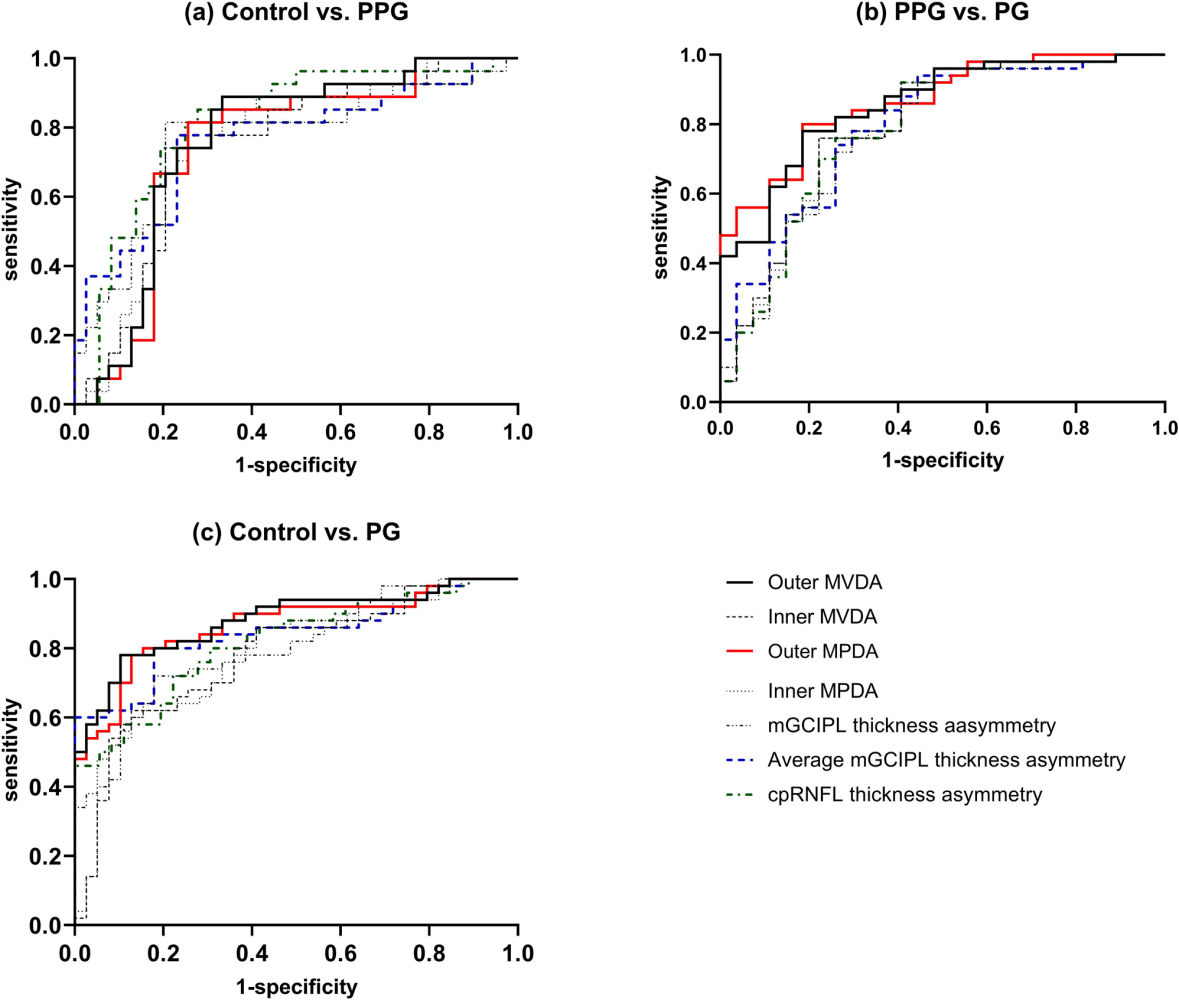
ROC curves of asymmetry of OMAG parameters and structural thickness parameters for differentiating between the groups.

Figure 2 demonstrated the diagnostic accuracy of vessel density and structural thickness asymmetry for differentiating between the following: (1) control and PPG, (2)PPG and PG, and (3) control and PG groups. For discriminating between healthy and PPG eyes, the highest AUC was obtained for cpRNFL thickness asymmetry (AUC = 0.81). Outer MPDA had the highest AUC for discriminating between PPG and PG eyes (AUC = 0.86), and outer MVDA had the highest AUC for discriminating between healthy and PG eyes (AUC = 0.88). AUC for asymmetry of all parameters decreased progressively with decreasing severity of glaucoma. We compared differences in AUC for vessel density asymmetry parameters and thickness asymmetry parameters between the groups (Table 4). No significant difference was found in the AUC of the asymmetry index of vessel density parameters and thickness parameters in terms of differentiating healthy eyes from those with glaucoma, regardless of whether they had PPG or PG. However, the AUC of outer MPDA was significantly higher than the AUC of average mGCIPL thickness asymmetry (p = 0.039) for differentiating between the PPG and PG groups.

**Table 4:** Comparisons of the diagnostic ability of asymmetry of OMAG parameters and structural thickness parameters for differentiating between the groups.

	P-value		
	Control vs PPG	PPG vs PG	Control vs PG
Macular outer vessel length density asymmetry vs Average mGCIPL thickness asymmetry	0.982	0.077	0.202
Macular outer vessel length density asymmetry vs cpRNFL thickness asymmetry	0.546	0.118	0.266
Macular outer perfusion density asymmetry vs verage mGCIPL thickness asymmetry	0.685	0.039	0.369
Macular outer perfusion density asymmetry vs cpRNFL thickness asymmetry	0.415	0.060	0.384

The p value: comparisons of AUC of the vessel density asymmetry parameters and structural thickness asymmetry parameters.

*AUC* area under the receiver operation characteristic curve, adjusted for age, gender, and spherical equivalence.

## Discussion

Through a simple method for defining asymmetry in this study, we found that OMAG macular asymmetry parameters were comparable with structural asymmetry parameters (mGCIPL) in differentiating either PPG or PG patients from controls. However, we found the discriminant capability of outer MPDA for differentiating between PPG and PG to be significantly better than that of average mGCIPL asymmetry. Macular vessel density has been shown to be reduced in glaucoma eyes in comparison with healthy eyes^5,18^. Rao et al. reported significantly lower diagnostic abilities for macular vessel density parameters in POAG than for peripapillary vessel density^19^. Most studies have concluded that the diagnostic ability of macular vessel density parameters in OCTA is moderate^18,20,21^. In support of such findings, our results revealed no significant difference in the macular vessel length or perfusion density between the control and PPG groups, regardless of the sector; however, the macular vessel length or perfusion density was only significantly lower in the PG group than in the control group. Previous studies have concluded that VF MD has a stronger correlation with OCTA parameters than with OCT parameters^7,8^. In this study, the superiority of outer MPDA to the asymmetry index of structural thickness in distinguishing PG from PPG may relate to the stronger correlation between VF and OCTA parameters.

Glaucomatous damage usually occurs in only one horizontal hemifield; therefore, the GHT of the VF, which measures disparity in retinal sensitivity between superior and inferior hemifield, is regarded as very useful in early glaucoma assessment^11,12^. However, the VF test is still a subjective and time-consuming test. We found that compared with structural asymmetry, outer MPDA had significantly higher diagnostic ability in PG than in PPG, whether through cpRNFL or mGCIPL measurement. To the best of our knowledge, no study has explored intraeye asymmetry of macular retinal vessel density between the upper and lower hemispheres. Although intraeye differences in macular retinal vessel density were not sensitive enough to identify very early glaucoma, such as PPG, in this study, they were able to differentiate PPG from PG. When PPG progresses to PG, only longitudinal follow-up with a VF test can confirm the diagnosis; thus, supplementary parameters such as vessel densities should be used in monitoring PPG. A longitudinal study found progressive macular vessel density loss in glaucoma eyes through serial OCTA measurements^22^. OCTA has even been reported to be useful in detecting changes in retinal microvasculature before VF damage becomes detectable in POAG patients^23^. Future studies with a larger sample size are needed to elucidate the clinical implications of macular vessel density asymmetry parameters measured using OCTA.

Measuring the inner retinal layers in the macular region is another strategy for the early diagnosis of glaucoma. No definite conclusion has been reached regarding whether the inner macular layer or cpRNFL is superior in differentiating glaucoma eyes from normal eyes. Mwanza et al. reported that average mGCIPL was comparable to average cpRNFL^24^, whereas Mahdavi et al. reported that average cpRNFL measurements were superior to global GCIPL thickness values for the detection of early glaucoma^25^. The AUC value from GCC thickness analysis was reported to range from 0.86 to 0.95^5,6^ for discriminating between normal and glaucoma eyes, and the range decreased to 0.6–0.8^3,26^ when the analysis was restricted to glaucoma eyes such as PPG eyes. The discriminant capability would not be the same in differentiating different stages of glaucoma. In our study, direct measurement of cpRNFL or mGCIPL thickness demonstrated significant differences between the control and PPG groups. However, cpRNFL thickness asymmetry was significantly higher in the PPG group than in the control group (p = 0.007), whereas mGCIPL thickness asymmetry showed no significant difference between the two groups. This might be because the definition of glaucoma was based on optic nerve appearance, which is in favour of peripapillary parameters. Studies have yielded inconsistent results regarding whether measurement of macular vessel density outperforms structural measurement of inner macular thickness in the diagnosis of glaucoma^5,6,20^. Leung et al.^27^ standardised the retinal layers for segmentation and the region of interest for analysis to enable a fair comparison between OCTA and OCT measurements. They concluded that the OCTA of the macula may have a limited role in the diagnostic evaluation of glaucoma. In this study, no significant difference was found in the macular vessel length or perfusion density between the control and PPG groups; however, mGCIPL thickness was significantly lower in the PPG group than in the control group. Our results are consistent with Leung’s finding that OCT structural measurement of inner macular thickness exhibited higher diagnostic performance in detecting glaucoma than did OCTA measurement of inner macular vessel density.

Macular hemifield differences in full retinal or inner retinal layer thickness have been reported to help in in early glaucoma diagnosis^10,13,28,29^. Posterior pole asymmetry analysis nstalled in the Spectralis SD-OCT (Heidelberg Engineering, Heidelberg, Germany) used a retinal full thickness value of 64 cells obtained from a macular area equivalent to a central 20° VF^30^. Um et al.^10^ and Seo et al.^29^ have used the Spectralis OCT with different approaches and attained similar results; specifically, that detecting asymmetry in macular hemifield full thickness may be a useful diagnostic aid in the detection of early glaucoma. Regarding inner retinal layers, Yamada et al.^13^ defined the asymmetry index based on a logarithmic ratio of upper to lower thickness and found that the ganglion cell layer asymmetry index for PPG (AUC = 0.81 for GCL; 0.79 for GCC) is a promising early indicator. In the present study, mGCIPL asymmetry was simply defined based on the ganglion cell analysis report provided by the Cirrus OCT, which measured an elliptical annulus centred at the fovea with outer vertical and horizontal axes of 4.0 and 4.8 mm, respectively. Although our measured area was smaller than that in the Spectralis or Optovue OCT machines, the AUC of average mGCIPL asymmetry for PPG (AUC = 0.76) and PG (AUC = 0.84) was still obtained. Furthermore, the commercially available analysis report by the Cirrus machine automatically divides the measured area into six parts. The superior or inferior mGCIPL that we used for asymmetry analysis was only related to the central part of the upper or lower hemisphere. In this study, by definition, average mGCIPL asymmetry covered larger areas than mGCIPL asymmetry; therefore, we used average mGCIPL thickness asymmetry instead of mGCIPL in the pairwise comparison. However, localised thinning of mGCIPL in PPG eyes might not have a marked effect on hemifield asymmetry as compared with control eyes. When mGCIPL progresses to loss in PG, this might substantially affect the mGCIPL asymmetry, which could explain why we found only mGCIPL thickness asymmetry to be significantly different between the control and PG groups but not between the control and PPG groups. This study has some limitations. First, because of its retrospective design, we could not investigate the blood pressure of patients or their antihypertensive medications. Most glaucoma patients were using IOP-lowering medications, and the effect of different IOP-lowering medications on OCT angiography imaging was unclear. Second, version 10.0 of our software did not provide for analysis of peripapillary vessel density. Additional details of optic disc vessel density would provide a comprehensive understanding of vascular asymmetry in glaucoma. Moreover, the ETDRS grid, which was used for OMAG macular sectoral measurements, was primarily developed for diabetic retinopathy evaluation rather than glaucoma evaluation. The GCIPL sectors were developed for the evaluation of structural changes in the macula in glaucoma. This may have biased the results in favour of the GCIPL measurements but our finding was that MPDA had a stronger ability to differentiate between PPG and PG comparing to GCIPL. Furthermore, the sector definitions of OMAG measurements used in the present study were the ones automatically demarcated by the software; hence, the asymmetry results would be applicable to all clinical settings when using this commercial software^21^. Third, we included patients with only mild to moderate VF defects in the PG group. Generalised loss, whether in nerve thickness or vessel density in both hemifields, usually occurs during glaucoma progression. Our findings might not apply to all glaucoma patients, especially not to those with severe VF defects. Fourth, we only excluded patients with diabetic retinopathy, and eight diabetic patients (three in the control group, three in the PPG group, and four in the PG group) did not have diabetic retinopathy in our study. A previous study reported that the density of vascular perfusion was reduced with increasing severity of diabetic retinopathy, even in non-diabetic retinopathy (NDR)^31^; however, Carnevali et al. investigated both superficial and deep macular vascular perfusion and reported only the deep capillary plexus in NDR was reduced^32^. Although only the superficial macular vascular perfusion was investigated in this study, the subclinical macular microvascular change in NDR might still be a confounding factor.

In conclusion, this study demonstrated that macular perfusion density asymmetry is not an aid in early glaucoma diagnosis for differentiating between healthy and PPG eyes; however, the discrimination ability of macular perfusion density asymmetry was significantly higher than structural asymmetry for differentiating between glaucoma patients with or without VF defects. Macular OCT angiography imaging could be included in the imaging algorithm for serial observation of patients with glaucoma. Longitudinal studies are needed to determine whether OCTA measurement can improve the detection or prediction of glaucoma progression and to elucidate the role of OCTA and VF in long-term glaucoma follow-up.
